# Activin A is upregulated by radiation in breast cancer cells and promotes conversion of CD4 T cells into regulatory T cells

**DOI:** 10.1186/2051-1426-1-S1-P119

**Published:** 2013-11-07

**Authors:** Claire Vanpouille-Box, Julie Diamond, Silvia Fomenti, Sandra Demaria

**Affiliations:** 1 NYU School of Medicine, Pathology Department, New York, NY, USA; 2 NYU School of Medicine, Radiation Oncology Department, New York, NY, USA

## 

Activin A is a member of the transforming growth factor beta (TGFβ) superfamily and a pleiotropic cytokine that regulates many processes, from reproduction and development to cancer and immunity. Activin A shares the smad2/3 signal transduction pathway with TGFβ and displays overlapping biological activities with the latter, including the ability to promote the differentiation of CD4 T cells to Th2 and regulatory T cells (Treg). Importantly, recent data indicate that activin A is expressed by some tumors, including breast cancer, suggesting that it could play a role in tumor escape from immune control. Radiotherapy (RT) delivered locally to a tumor induces the development of anti-tumor T cells, but its pro-immunogenic effects are hindered, in part, by concomitant activation of latent TGFβ and increase of Treg. While different mechanisms have been implicated in RT-induced Treg increase, the pathways responsible for this effect remain unclear. Here we tested the hypothesis that activin A is upregulated by RT in breast cancer cells and contributes to the generation of adaptive Treg. Three mouse breast cancer cell lines, 67NR, TSA and 4T1, which represent tumors of decreasing immunogenicity and increasing metastatic ability, were used. Expression of inhibin A (Inhba, the gene encoding activin A) was determined by qPCR. Secretion of activin A by untreated and irradiated tumor cells exposed to single dose (6Gy, 8Gy, 12Gy and 20Gy) or multifraction (5x6Gy; 3x8Gy) RT was quantified by ELISA. Transwell co-culture was used to assess the ability of activin A released by irradiated cancer cells to convert naïve CD4 T cells into Treg. While 67NR, TSA and 4T1 cells expressed comparable levels of Inhba mRNA, only the most aggressive and metastatic 4T1 cells produced high levels of activin A (67NR: 37.1; TSA: 8.1; 4T1: 448.6 pg/mL for 10^5^ cells/24h). RT significantly increased activin A secretion, with the largest increase seen after 3x8Gy RT regimen (67NR: 85.8; TSA: 55.1; 4T1: 993.1 pg/mL for 10^5^ cells/24h; p<0.05). Conversion of naïve CD4+ T cells into Treg upon activation in the presence of irradiated 4T1 cells was markedly enhanced (Control: 8.1%, irradiated 4T1: 46.9% of Treg). This effect was partially reversed in the presence the activin A inhibitor follistatin (23.8% of Treg). Data suggest that increased activin A secretion by breast cancer cells may contribute to the enhanced generation of Treg in irradiated tumors. Experiments are ongoing to determine whether blocking activin A improves immune-mediated rejection of irradiated tumors in vivo. Supported by DOD BCRP Post-doctoral fellowship W81XWH-13-1-0012.

